# Role of infection in urea cycle disorders

**Published:** 2014-02

Ornithine transcarbamylase deficiency (OTCD) is a type of urea cycle disorder – a disease in which the body fails to clear toxic ammonia from the bloodstream. The accumulation of ammonia in the blood (hyperammonaemia) can lead to potentially life-threatening metabolic disturbances in affected individuals, known as acute metabolic decompensation. Several factors, including infection, are thought to precipitate these metabolic aberrations. In this study, Peter McGuire and colleagues identify infection as the most common cause of acute decompensation in a prospective cohort of individuals with OTCD. To explore the metabolic changes that occur, they developed an experimental mouse model in which decompensation with hyperammonaemia is triggered by influenza infection. In response to infection, mice demonstrated altered hepatic immune function and reductions in urea cycle enzyme activity and urea cycle intermediates. These findings could help guide the development of new approaches to manage acute metabolic decompensation in OTCD and related inborn errors of metabolism. Page 205

